# Perceptions of two different alcohol use behaviours in pregnancy: an application of the prototype/willingness model

**DOI:** 10.1080/21642850.2022.2143362

**Published:** 2022-11-07

**Authors:** Tess Fletcher, Barbara Mullan, Amy Finlay-Jones

**Affiliations:** a FASD Research Australia Centre for Research Excellence, Telethon Kids Institute, Nedlands, Australia; b Enable Institute, Curtin University, Perth, Australia; c School of Population Health, Curtin University, Perth, Australia

**Keywords:** Prevention, health behaviour, prenatal alcohol exposure, pregnancy

## Abstract

**Objective:**

This study explored whether exposure to either an ‘ambiguous consumption’ prototype (no amount of alcohol specified) or a ‘small consumption’ prototype (‘small’ amount of alcohol specified) had an impact on prototype perceptions of, and willingness to use, small amounts of alcohol during pregnancy.

**Method:**

Participants were 140 women living in the UK, aged 20–45 years old, of whom 92% had previously been, or intended to become, pregnant. Participants completed measures to assess how favourably they viewed alcohol use in pregnancy, how similar they felt to those who would use alcohol in pregnancy and how responsible they perceived those who would use alcohol in pregnancy to be. Participant’s own willingness to use small amounts of alcohol during pregnancy was also assessed.

**Results:**

Having at least one child was positively correlated with being willing to accept an alcoholic drink while pregnant for those exposed to the ‘ambiguous consumption’ prototype only. Although perceptions of favourability, similarity and responsibility were consistently higher for those exposed to a ‘small consumption’ condition, there were no differences in willingness to drink according to exposure.

**Conclusion:**

This research suggests that women’s perceptions of those who drink alcohol while pregnant differ according to the amount of alcohol that they perceive as typical. This may have implications for how alcohol use in pregnancy is portrayed in public health messaging, particularly regarding the level of acceptance that may be associated with low to moderate alcohol use.

## Introduction

### Alcohol use during pregnancy

Alcohol use during pregnancy is an ongoing public health concern due to the risk prenatal alcohol exposure poses to a foetus’ development (Williams & Smith, 2015). With no known safe level of exposure, many health agencies and governments take a precautionary approach by recommending no alcohol use during pregnancy (Department of Health, 2016; National Health and Medical Research Council, 2020). Women receive much of their information and guidance about alcohol use and pregnancy from health professionals as well as from family and friends (Anderson et al., 2014; Tsang et al., 2020). However, the quality and veracity of this information can vary between sources and can be contradictory (Anderson et al., 2014; Elek et al., 2013), with one particular area of confusion being the level of risk associated with low to moderate alcohol use during pregnancy.

### Confusion about ‘safe’ limits

The inconsistent evidence for harm associated with low to moderate levels of alcohol use (Comasco et al., 2018) can cause hesitation in recommending abstinence by some health professionals, as they may be concerned about alarming women unnecessarily (Coons et al., 2017b). Additionally, binge drinking and excessive drinking are often considered by health professionals as being of the most concern, whereas low to moderate use is often seen as inconsequential (Coons et al., 2017a). This belief that abstinence is not always completely necessary may filter down to women who are perceived as being at low risk and therefore do not receive the abstinence message. Furthermore, the definition of low, moderate, and high levels of alcohol use is inconsistent (O’Leary & Bower, 2012) and often linked to known risks, such as with commonly used screening tools (Bush et al., 1998). This inconsistency may lead to further confusion among the public with regard to risk and what constitutes a ‘safe’ level of use, if any.

### Determinants of alcohol use including prior use and social influences

Prior alcohol use is associated with the likelihood a woman will consume alcohol while pregnant. For example, alcohol use during a prior pregnancy is associated with intentions to drink alcohol while pregnant (Peadon et al., 2011), while levels of alcohol use before conception are predictive of alcohol use during pregnancy (Skagerstrom et al., 2011). Drinking alcohol is a common aspect of social life in many countries and can be a barrier to abstaining from alcohol in general (Pennay et al., 2018) as well as during pregnancy (France et al., 2013; Gouilhers et al., 2019; Loxton et al., 2013). Prior research suggests that alcohol use decisions while pregnant may be influenced by social norms and pressures (Loxton et al., 2013; Meurk et al., 2014). In particular, drinking alcohol on special occasions, such as weddings or celebrations, is a commonly reported behaviour amongst those who otherwise tend to support or engage in alcohol avoidance during pregnancy (Muggli et al., 2016; Tsang et al., 2021). Given that women must navigate conflicting messages about risk, and social pressure to both drink and to avoid stigma, it is important to explore women’s intentions regarding alcohol use from within the context of their social perceptions and experiences.

### What is ‘alcohol use in pregnancy’?

An additional complexity in identifying determinants of alcohol use in pregnancy is the framing used when asking about alcohol use and related beliefs, intentions, and perceptions. For example, research has found that although women widely reported supporting abstinence in pregnancy, ‘moderate’ consumption of alcohol was also seen as reasonable (Fletcher et al., 2021; Hammer & Inglin, 2014). These conflicting perspectives may be due to the level and frequency of alcohol use which women use as a reference point when responding to questions about alcohol use in pregnancy. These different reference points may have implications for how women interpret advice about alcohol in pregnancy, for example if women do not consider having a sip of champagne at a wedding as ‘alcohol use in pregnancy’ they may feel that they are behaving in accordance with the guidelines if they were to do so. Furthermore, if they perceive themselves as drinking at a ‘low’ level, they may feel that the advice about not drinking alcohol in pregnancy does not apply to them but rather is intended for those who they perceive as being at greater risk of harm. This kind of thinking may be exacerbated by the stigma that surrounds alcohol use in pregnancy (Corrigan et al., 2018), with individuals who support alcohol use in ‘moderation’ also expressing judgemental beliefs about those who they perceive as drinking in pregnancy at a level that requires behaviour change, thus, creating a cognitive dissonance between their own casual use and that which can be considered categorically harmful. It is therefore important to determine whether these reference points influence how women perceive the behaviour of alcohol use in pregnancy and whether this impacts their willingness to engage in the same behaviour.

### Prototype/willingness model

The prototype/willingness model (Gibbons et al., 1995; Gibbons et al., 1998) is a social cognitive theory that explores the role that perceptions of the ‘typical person’ (i.e. prototypes) play in someone’s willingness to enact certain behaviours. When thinking of a particular behaviour, an individual has a perception of the typical person who would engage in that behaviour and their associated traits or attributes (Lettow et al., 2013). The prototype/willingness model states that perceptions of a prototype predict an individual’s willingness to engage in the same behaviour given the opportunity (Rivis et al., 2006). That is, if someone holds certain perceptions of the prototypical person engaging in a behaviour, they would expect their peers to hold the same views, which in turn would make them more or less willing to engage in similar behaviour (Norman et al., 2007). One such perception is the perceived favourability of a prototype; that is how favourably or unfavourably one sees the prototype. In addition to perceptions of favourability, an individual can also reflect on the extent to which they identify themselves as similar or dissimilar to the prototypical person. The more similar to the prototype they see themselves as being, the greater impact that favourability is expected to have (Rivis et al., 2006). For example, holding a favourable prototype about an individual who smokes is more likely to result in an individual smoking if they also see themselves as similar to someone who would smoke.

Alcohol use during pregnancy appears to be dichotomised into ‘risky’ alcohol use and ‘safe’ alcohol use by both health professionals and broader society despite any evidence of a safe level of use (Bagley & Badry, 2019; Fletcher et al., 2021). This is likely to have implications for the level of risk that is perceived, that is if one believes that there is a ‘safe’ level of alcohol use they may be more likely to perceive the behaviour of low to moderate alcohol use in pregnancy as being responsible and risk-free. Although not explicitly mentioned in the prototype/willingness model, perceived responsibility may be a relevant aspect of how the behaviour of alcohol use in pregnancy is constructed in the public eye (Hammer & Inglin, 2014). Additionally, an emphasis on the harm associated with heavy alcohol use may compound this distinction between what constitutes a safe/responsible and a risky/irresponsible behaviour and contribute to furthering stigmatising beliefs (Coons et al., 2017).

Importantly, prior research into alcohol use behaviour in pregnancy has many inconsistencies with regard to what is considered ‘no exposure’ (e.g. up to 2 drinks) and what is considered ‘alcohol use’ (Popova et al., 2017). Furthermore, qualitative research into women’s attitudes and beliefs does not typically discriminate what exactly the behaviour of alcohol use in pregnancy being studied entails (Meurk et al., 2014). Considering the lack of consistency in how alcohol use in pregnancy is portrayed, interpreted, and studied, it is unknown whether the extent to which the associated stereotypes about the typical person who drinks alcohol while pregnant varies according to specific alcohol use behaviours. Additionally, whether these stereotypes impact an individual’s willingness to also consume alcohol while pregnant is also unknown. To further explore this possible distinction, it is necessary to conduct research that clearly outlines or documents the behaviour being studied and to establish whether willingness to engage in alcohol use during pregnancy differs according to perceptions of the typical person who drinks while pregnant.

## The present study

The aim of the present study was to use the prototype/willingness model to compare prototype perceptions between participants exposed to two different prototypes and to explore willingness to use small levels of alcohol during pregnancy according to exposure conditions. The two prototypes were ‘ambiguous consumption’ (no amount of alcohol specified) and the ‘small consumption’ prototype (‘small’ amount of alcohol specified). Three prototype perceptions were examined, the perceived favourability of the prototype, the respondent’s perceived similarity to the prototype, and the perceived responsibility of the prototypes.

It was hypothesised that:

H1: Respondents in the ‘small consumption’ condition would perceive prototypes to be higher in similarity, responsibility, and likeability than those in the ‘ambiguous consumption’ condition.

H2: Prototype perceptions would have a strong positive relationship with self-reported willingness to drink a small amount of alcohol during pregnancy.

H3: Exposure to the ‘small consumption’ prototype would have a stronger positive correlation with willingness to drink a small amount of alcohol during pregnancy than the ‘ambiguous consumption’ prototype.

## Method

### Participants and procedure

Participants in this study were 140 people aged between 20 and 45 years who identified as women, lived in the UK, and consumed alcohol. Participants were recruited through the paid recruitment platform Prolific and completed an online survey by providing their informed consent and demographic data, including age, marital status, education, employment status, and pregnancy history. Participants were then presented with the definition of a prototype, taken from Gibbons et al. (1995, p. 85): ‘The following questions concern your images of people. What we are interested in here are your ideas about typical members of different groups. For example, we all have ideas about what typical movie stars are like or what the typical grandmother is like. When asked, we could describe one of these images — we might say that the typical movie star is pretty or rich, or that the typical grandmother is sweet and frail. We are not saying that all movie stars or all grandmothers are exactly alike, but rather that many of them share certain characteristics’. Participants were randomised to one of two conditions, whereby they were presented with a prompt to consider either the ‘typical person who drinks alcohol in pregnancy’ (‘ambiguous consumption’ condition) or the ‘typical person who drinks a **small** amount of alcohol in pregnancy’ (‘small consumption’ condition). They were then asked to provide three to five words that best describe that kind of person. This was used to prime participants with a clear image of the typical person who engages in the behaviour. Respondents were primed to think of one of two different ‘types’ of behaviours, i.e. a small level of alcohol use in pregnancy and an ambiguous level of alcohol use in pregnancy, as they completed the measures in the survey. Following survey completion, participants were paid GB£9.6 per hour for their time.

## Measures

### Pregnancy intentions

Participants were asked whether they intended to become pregnant in the future (<2, 3-5, 5 < years).

### Alcohol consumption

Participants were asked when they last had a drink containing alcohol (never, a week or less ago, between 2–4 weeks ago, between 1–3 months ago, over 3 months ago and prefer not to say).

### Prototype perceptions

Following randomisation to either the ‘ambiguous consumption’ or ‘small consumption’ conditions, participants were asked to assign a rating on a 5-point Likert scale of how likeable they thought the prototype individual was (from ‘extremely unlikeable’ to ‘extremely likeable), how responsible they believed they were (from ‘extremely responsible’ to ‘extremely irresponsible’) and how similar they felt they were to them (from ‘not at all similar’ to ‘very similar’). Although not originally part of the prototype/willingness model, responsibility was included as an additional prototype perception due to it being commonly referenced in the literature about alcohol use in pregnancy (Hammer & Inglin, 2014; Lyall et al., 2021).

### Subjective measure of alcohol use in pregnancy

Participants in the ‘ambiguous consumption’ were asked to specify how much alcohol they thought the typical person who drinks alcohol in pregnancy would have, including the amount of alcohol, type of alcohol, and frequency of use. Conversely, those in the ‘small consumption’ condition were asked to specify how much alcohol someone who had a small amount would typically drink.

### Willingness to consume small amounts of alcohol in pregnancy

Two items were used to assess the respondents’ willingness to consume alcohol in pregnancy when provided the opportunity. The respondents were presented with two hypothetical scenarios and asked to indicate, along a 7-point Likert scale, how willing (from ‘not at all willing’ to ‘very willing’) they would be to accept and finish a glass of champagne at a wedding and how willing they would be to say no and refuse the offer of a glass of wine at dinner with a friend. These scenarios were chosen as they reflect situations in which women report being inclined to or actually consuming alcohol while pregnant (Fletcher et al., 2021; Tsang et al., 2021). Responses to item two (willingness to refuse the drink) were reverse coded and added together with responses to item 1. This score was then divided by two to create a composite measure of willingness to consume a small amount of alcohol while pregnant (N.B. willingness).

## Ethical approval

The study was approved by the Curtin University Human Research Ethics Committee (HREC number HRE2019-0339)*.*

## Results

### Demographics

Of the whole sample (N = 140), 54% were aged 20–30 years old (*M* = 30.81, *SD* = 7.482). The majority (69%) had completed either an undergraduate or a postgraduate degree and 74% were employed either part-time or full-time. Additionally, 90% drank alcohol within the last month (86% ‘ambiguous’, 94% ‘small’), 40% had previously been pregnant (44% ‘ambiguous’, 36% ‘small’) and 59% intended to become pregnant in the future (56% ‘ambiguous’, 62% ‘small’). Six percent of participants had never been and did not ever intend to become pregnant. Additional demographics are outlined in Table 1. An a priori power analysis was conducted using G*Power version 3.1.9.7 (Faul et al., 2007) to determine the minimum sample size required. Results indicated the required sample size to achieve 80% power for detecting a medium effect, at a significance criterion of α = .05, was N = 64 for each group. As such the study was adequately powered.

**Table 1.** Demographic Characteristics (N = 140).

Demographic measure	‘Ambiguous’ N = 71		‘Small’ N = 69	
	N	%	N	%
*Age*				
20–25	19	26.8	25	36.2
26–30	17	23.9	15	21.7
31–35	14	19.7	9	13.0
36–40	10	14.1	9	13.0
41–45	11	15.5	11	15.9
*Marital status*				
Single, never married	31	43.7	36	52.2
Married/de facto	37	52.1	31	44.9
Divorced	2	2.8	0	0.0
Separated	0	0.0	1	1.4
Prefer not to say	1	1.4	1	1.4
*Education - highest level*				
Secondary education	5	7.0	5	7.2
High school diploma	15	21.1	11	15.9
Technical/Community College	5	7.0	2	2.9
Undergraduate degree	30	42.3	39	56.5
Graduate degree	15	21.1	12	17.4
Doctorate degree	1	1.4	0	0.0
*Employment status*				
Employed full-time	43	60.6	41	59.4
Employed part-time	11	15.5	9	13.0
Unemployed looking for work	2	2.8	5	7.2
Stay at home parent	3	4.2	3	4.3
Student	11	15.5	9	13.0
Disability	1	1.4	1	1.4
Prefer not to say	0	0.0	1	1.4
*Pregnancy history*				
Currently pregnant: No	71	100.0	69	100.0
Previously pregnant: Yes	31	43.7	25	36.2
Biological children: Yes	24	33.8	17	24.6
*Pregnancy intention*				
Yes, within the next 2 years	17	23.9	14	20.3
Yes, within the next 5 years	11	15.5	14	20.3
Yes, in 5 years or more	12	16.9	15	21.7
No, never	15	21.1	17	24.6
Unsure	16	22.5	9	13.0
*Last drink of alcohol*				
A week or less ago	46	64.8	44	63.8
Between 2–4 weeks ago	15	21.1	21	30.4
Between 1–3 months ago	4	5.6	2	2.9
Over 3 months ago	6	8.5	2	2.9

### Prototype characteristics

Respondents generated 278 characteristics to describe the typical person who would drink an ambiguous amount of alcohol in pregnancy and 268 characteristics to describe the typical person who would drink a ‘small’ amount of alcohol during pregnancy. The characteristics reported by at least 10% of the sample are outlined in Table 2.

**Table 2.** Words used to describe each prototype (N = 140).

Prototype Exposure					
‘Ambiguous’ N = 71			‘Small’ N = 69		
Characteristic	N	%	Characteristic	N	%
Irresponsible	30	42%	Selfish	16	23%
Selfish	24	34%	Irresponsible	16	23%
Careless	13	18%	Risky/risk-taker	15	22%
Uneducated	11	15%	Careless	10	14%
Addict	7	10%	Normal	8	11%

### Descriptive statistics

The correlations between variables for both prototype conditions are presented in Tables 3 and 4, along with means and standard deviations. Income and willingness to refuse a drink while pregnant were not significantly correlated with any of the other main variables under either condition. The mean prototype likeability, responsibility and similarity ratings were well below the scale mid-point for both groups. A Fisher’s z-test was used to compare the significance of the difference between correlations for each group (Fisher, 1921; Soper, 2022). Of all the correlations, the only significant difference between groups was for similarity and responsibility with the relationship being significantly stronger for the ‘small’ group compared to the ‘ambiguous’ group, *z *= 2.909, *SEM *= 0.085, *p = *0.004 (two tails).

**Table 3.** Descriptive statistics and correlations between the study variables for ‘ambiguous consumption’ prototype (N = 71).

	AGE	CHILD.	LIK.	RES.	SIM.	ACC.	REF.	WILL.	Mean	SD
Age	1	.511*	0.20	0.05	0.12	.285	0.07	0.21	31.21	7.45
Children		1	.387*	0.21	0.21	.340*	−0.06	0.13	N/A	N/A
Likability			1	.445*	.478*	.384*	0.01	0.20	1.97	0.83
Responsibility				1	.495*	.429*	−0.06	0.17	1.68	0.98
Similarity					1	.664*	0.13	.449*	1.76	1.1
Accept						1	0.05	.555*	1.99	1.62
Refuse							1	−0.859	4.79	2.63
Willingness								1	2.59	1.58

Note. Children (Child.), Likability (Lik.), Responsibility (Res.), Similarity (Sim.), Accept (Acc.), Refuse (Ref.), Willingness (Will.), * *p *< 0.01.

**Table 4.** Descriptive statistics and correlations between the study variables for ‘small consumption’ prototype (N = 69).

	AGE	CHILD.	LIK.	RES.	SIM.	ACC.	REF.	WILL.	Mean	SD
Age	1	.616*	.263	0.09	0.11	0.12	−0.07	0.02	30.41	7.54
Children		1	0.17	0.13	0.13	0.17	−0.17	−0.04	N/A	N/A
Likability			1	.653*	.680*	.521*	−0.08	.244	3.01	1.02
Responsibility				1	.780*	.623*	−0.02	.353*	2.6	1.27
Similarity					1	.725*	0.04	.456*	2.46	1.45
Accept						1	0.04	.616*	2.41	1.93
Refuse							1	-.810*	4.57	2.56
Willingness								1	2.92	1.64

Note. Children (Child.), Likability (Lik.), Responsibility (Res.), Similarity (Sim.), Accept (Acc.), Refuse (Ref.), Willingness (Will.), * *p *< 0.01.

### Group differences

A series of independent samples *t*-tests were run to compare the ratings of likeability and individual willingness to consume small amounts of alcohol in pregnancy reported by those in the ‘ambiguous consumption’ condition (N = 71) compared to the ratings of those in the ‘small consumption’ condition (N* *= 69). A series of Welch’s *t*-tests were also run to determine if there were differences in ratings of similarity and likeability for those in both conditions due to the assumption of homogeneity of variances being violated, as assessed by Levene’s test for equality of variances (similarity, *p *= .001; responsibility, *p* = .001). Although the Shapiro–Wilk statistic was significant for all variables, upon examination of the histograms and QQ plots the data appeared to be normally distributed except for the scores for perceived responsibility. However, given that large and relatively equal sample sizes (N* >* 30-40) are robust against violations of the assumption of normality, the results were interpreted as having satisfied the assumption. As four t-tests were run a Bonferroni correction was applied and the results were interpreted at a significance value of 0.01. Table 5 outlines the findings of the independent samples and Welch’s *t*-tests.

**Table 5.** t-test results comparing prototype exposure, prototype perceptions and willingness.

	‘Ambiguous’		‘Small’		*T (*df)	*p*
	M	SD	M	SD		
Likeability	1.97	0.83	3.00	1.01	6.58 (138)	<.001
Similarity	1.76	1.10	2.43	1.45	3.09 (126)	0.002
Responsibility	1.68	0.98	2.60	1.27	4.80 (126)	<.001
Willingness	1.60	1.58	2.93	1.63	1.24 (138)	0.218

The *t-test* for likeability was statistically significant with those exposed to the ‘ambiguous consumption’ prototype (*M* = 1.97, *SD* = 0.83) rating the prototype as less likable than the group exposed to the ‘small consumption’ prototype (*M* = 3.00, *SD* = 1.01). For similarity, the Welch’s *t*-test was statistically significant with those exposed to the ‘ambiguous consumption’ prototype (*M* = 1.76, *SD *= 1.10) rating themselves as less similar to the prototype than the group exposed to the ‘small consumption’ prototype (*M* = 2.43, *SD* = 1.45). Similarly, the Welch’s *t*-test was statistically significant for responsibility with those exposed to the ‘ambiguous consumption’ prototype (*M* = 1.68, *SD *= 0.98) rating the ‘ambiguous consumption’ prototype as less responsible than the group exposed to the ‘small consumption’ prototype (*M* = 2.60, *SD* = 1.27). There were no statistically significant differences in willingness to refuse or accept a drink while pregnant as a function of prototype exposure.

## Discussion

In this study we explored whether exposure to one of two drinker prototypes, ambiguous consumption, and small consumption, was related to perceptions of those who drink alcohol during pregnancy and individual willingness to use small amounts of alcohol during pregnancy. For those exposed to the ‘ambiguous consumption’ prototype, having at least one child was positively correlated with being willing to accept a drink while pregnant. This finding was not present for the ‘small consumption’ condition. Given the relatively recent move towards recommendations for abstinence in the UK and the associated public health efforts, it could be expected that those of younger age may have been exposed to that messaging earlier and more consistently (Department of Health, 2016).

The findings of this study support the first hypothesis that ratings of favourability, similarity, and responsibility would be higher for those exposed to the ‘small consumption’ prototype as opposed to the ‘ambiguous consumption’ prototype. This is not surprising given that research into alcohol and pregnancy has documented wide-spread stigma surrounding alcohol use in pregnancy (Corrigan et al., 2018; Eguiagaray et al., 2016). For both conditions, similarity was significantly correlated with willingness to consume alcohol while pregnant. This suggests that if someone has friends or influencers that they see as similar to themselves, who drink while they are pregnant, they may be more willing to also drink while pregnant. Conversely, abstinence messages may need to be delivered by a relatable figure that people perceive as likeable and similar to increase the likelihood that others will be willing to engage in abstinence behaviours themselves. Due to the ambiguity of the behaviour, it was expected that there would be greater variation in how individuals interpreted the ‘ambiguous consumption’ prototype versus the ‘small consumption’ prototype in terms of the amount of alcohol consumed. However, there was less variation and lower ratings in similarity scores under the ambiguous condition than the small condition suggesting that participants in this condition interpreted the behaviour similarly. This may have made the effect of perceived similarity to be more pronounced as scores were skewed to the negative and more universal within the ambiguous group. Additional exploration of this finding would be needed to make any further inferences.

Interestingly, perceived responsibility was positively correlated with willingness to consume a small amount of alcohol while pregnant for the ‘small consumption’ condition but not the ‘ambiguous consumption’ condition. Potentially, there was a floor effect such that there was little variability in the perceived responsibility of those who engage in the ambiguous behaviour of drinking alcohol while pregnant so a relationship with willingness could not be detected. Additionally, in opposition to expectations, the ‘small consumption’ condition could have actually left more room for interpretation and, therefore, greater variability in perceptions of responsibility. That is, participants may have differed in the reference point they were using to compare a small amount of use to; for example, some people may have been comparing a ‘small amount’ to ‘total abstinence’ (ergo, irresponsible) while others may have compared a ‘small amount’ to ‘a lot’ (ergo, responsible).

Due to the open-ended nature of the question about the typical amount of alcohol consumed by each prototype, it is difficult to quantify any differences in the amount of alcohol underpinning the perceptions of the alcohol use behaviours for participants in each condition. For example, responses covered a broad range including from ‘a glass of prosecco on an evening out or special occasion’ to ‘binge drinking, e.g. once a week, likely vodka, cheap wine, gets really drunk’ for those in the ‘ambiguous consumption’ condition and from ‘a sip of wine on special occasions - not more than once per fortnight’ to ‘A glass of wine a day throughout their pregnancy’ for those in the ‘small consumption’ condition. However, the fact that the small amount of consumption was rated more positively overall supports the assumption that a small amount of alcohol was likely perceived as lower than the ambiguous amount. Additionally, data were collected in this way so that the range of responses could be used to develop a more precise question for further research. That is, these findings will inform the development of a question that allows for the standardised measurement of the type, amount, and frequency of alcohol use and the calculation of the amount of alcohol consumed per week/maximum amount on any given day.

Although there were differences in perceptions of the prototypes, there was a lack of a difference between the groups regarding their willingness to consume a small amount of alcohol while pregnant. Therefore, the final hypothesis was not supported. Other studies have also found limited to no effect due to prototype manipulation; for example, there was no effect on the willingness of female undergraduates to binge drink as a result of prototype manipulation (Todd & Mullan, 2011). Further research is needed to explore the role that manipulating prototypes may play in altering prototype perceptions and willingness and in prompting or facilitating behaviour change (Davies & Todd, 2021).

The findings of this research indicate that there are distinctions in how the ambiguous behaviour of alcohol use during pregnancy is perceived as opposed to ‘small amount’ use during pregnancy. However, highlighting these distinctions does not appear to be related to willingness to drink a small amount while pregnant. Further research should explore the specific beliefs that underlie decisions regarding alcohol use during pregnancy. Although willingness to drink a small amount while pregnant does not appear to be related to perceptions of the prototypical drinker, further research should explore whether the reasoned pathway reflecting intentions to drink differs according to the level of alcohol use being studied.

## Strengths and limitations

This study allowed for respondents to nominate characteristics that they felt reflected the stereotypical person who drinks alcohol instead of asking participants to respond to pre-determined characteristics. For a sensitive and highly stigmatised issue such as alcohol use in pregnancy, this approach meant that participants were able to spontaneously nominate characteristics and thus provided an ethically responsible way to collect these data. The use of an online panel for recruitment may also have had implications for the generalisability of the findings however, a meta-analysis of comparisons between field data and online panel data did not find meaningful differences in the validity and reliability of the data collected. Thus, suggesting that online panels are an appropriate recruitment method.

Although there were significant differences between how the two behaviours were perceived, there was little discernible impact on whether participants would be willing to drink alcohol while pregnant. One potential reason for this lack of relationship could be how willingness was measured in this study. Although the willingness questions were devised to represent common scenarios in which a pregnant person may have to make a momentary decision about whether to drink, they may have been too specific. Additionally, the use of two different scenarios for inclusion as a composite may have complicated the measure.

Another reason for the lack of a relationship may be due to the inclusion of those who did not intend to become pregnant in the future. Additional research in this space could explore the willingness of those who intended to become pregnant in the future and could stratify the sample according to past pregnancy history. Given a larger sample size, the sample could also be stratified according to different levels of current alcohol use to determine the impact on prototype perceptions and willingness. Additionally, it may be important to explore the specific contexts in which individuals drink, to then identify whether perceptions of prototypical alcohol consumers and individual willingness differed between those with high rates of social drinking as compared to those drinking in private or not regularly.

An additional limitation in regard to measurement of willingness in this study may be the requirement placed on participants to deliberate on questions about alcohol use in pregnancy prior to measuring willingness. It has previously been suggested that the momentary aspects of willingness are not being sufficiently accounted for when conducting studies using the prototype/willingness model as a theoretical framework (Davies & Todd, 2021). However, in lieu of a real-life social situation, it was necessary for participants in this study to be prompted to consider their perceptions of the behaviour prior to stating their willingness to engage in the behaviour of alcohol use in pregnancy. Particularly as this research was focussed on the role that the different perceptions of alcohol use behaviours played in individual’s willingness. However, as suggested by Davies and Todd (2021) future research should explore novel techniques, such as measuring implicit attitudes (Davies et al., 2017; Ratliff & Howell, 2015), to better approximate the conditions in which the prototype/willingness model is expected to be a useful model of behaviour. This type of method could be particularly useful for an ethically complicated area such as alcohol use during pregnancy.

## Conclusion

The results of this study indicate that people perceive those who drink alcohol while pregnant differently depending on their perceived consumption level. This may have implications for health promotion messaging because although people may not approve of drinking alcohol while pregnant, they may be more accepting of what they perceive to be a small or ‘low-risk’ amount. However, the amount that constitutes a ‘small amount’ of alcohol is subjective and open to interpretation. More clarity about what people perceive the behaviour of ‘drinking alcohol during pregnancy’ to entail is necessary to better understand people’s willingness to do so.

## Data Availability

The data that support the findings of this study are available from the corresponding author, TF, upon reasonable request.
